# Journalistic Notes

**Published:** 1891-06

**Authors:** 


### Journalistic Note.

---

The American Microscopical Journal is heartily welcome to our exchange table. It retains the same size and form given it by its founder, Prof. Hitchcock. As the *Journal* contains much of value, we shall look forward to each month's arrival with considerable interest.

